# External fixators and lengthening systems in pediatric upper limb

**DOI:** 10.1177/18632521251327127

**Published:** 2025-03-17

**Authors:** Nunzio Catena, Chiara Arrigoni, Marcos Carvalho, Ida Matic, Sebastian Farr

**Affiliations:** 1 Hand Surgery and Reconstructive Microsurgery Unit, IRCCS Istituto Giannina Gaslini, Genova, Italy; 2 Department of Pediatric Orthopedics, Pediatric Hospital, Centro Hospitalar e Universitário de Coimbra (CHUC), EPE, Coimbra, Portugal; 3 Children’s Hospital Zagreb, Zagreb, Croatia; 4 Department of Pediatric Orthopedics and Foot and Ankle Surgery, Orthopedic Hospital Speising, Vienna, Austria

**Keywords:** External fixator, upper limb, pediatric deformities

## Abstract

The use of external fixators is part of the cultural background of orthopedic surgeons in treating numerous clinical conditions. Over the years, fixator design and biomechanical knowledge have led to different solutions and techniques, and bone lengthening and its better understanding come together with the development of external fixators and the application of the biological principle of distraction osteogenesis. The authors conducted a literature review about using external fixators and lengthening systems in pediatric upper limbs. Despite the applications of external fixators in upper limbs remaining much more limited than those of the lower limbs, there are indications of traumatic, congenital, tumor, and infectious etiologies. However, despite the spread of new systems of plate and screws and intramedullary lengthening nails, the problems about when to use external fixation remain unsolved. Another debated point is about using monolateral or circular frames for humeral lengthening and the correction of forearm deformities in multiple hereditary exostoses disease (MHE) or radial longitudinal deficiency sequelae. Monoaxial fixators retain a prominent role for skeletal lengthening in all the districts examined, although their role could be outclassed by the motorized intramedullary nails, especially for humeral lengthening. Hexapod systems are likely to represent the future for the correction of multiplanar forearm deformities; however, multicenter studies on larger series will be necessary to better validate their applications and advantages.

## Introduction

The use of external fixators is part of the cultural background of orthopedic surgeons in treating numerous clinical conditions.

Historically, external fixation has been used to treat fractures, not only as a provisional treatment or as part of a damage control strategy (open fractures with significant soft tissue compromise or bone loss or high-energy fractures in hemodynamically unstable patients) but also as an option for definitive treatment.

With the growing interest in external fixators, new indications have emerged, not only in trauma but also in tumor pathology, congenital or acquired deformities, or limb length discrepancies following trauma or infection.^1–3^

Over the years, fixator design and biomechanical knowledge have led to different solutions and techniques; bone lengthening and its better understanding come together with the development of external fixators and the application of the biological principle of distraction osteogenesis.

Nowadays, because of creativity and technological advances in biomedical engineering, there are several different types of external fixators including uniplanar, multiplanar, unilateral, bilateral, and circular, and their applicability varies widely depending on the intended use.^4^

Despite the wider applications of external fixators in lower limbs, moreover, there are several indications in the upper extremity, including traumatic, congenital, tumor, and infectious etiologies although the spread of new systems of plate and screws and intramedullary lengthening nails are reducing their use.

In this article, we explore the use of external fixators and intramedullary lengthening devices for several indications in the pediatric upper extremity, although which kind of device is more reported in each condition is still another debated point. We approach the issue in three items: lengthening, axis correction, and surgery related to complications.

Moreover, while considering its use as a safe and minimally invasive procedure, in pediatric patients and elective surgery, its application should be carefully discussed with the patient and/or family and, whenever possible, considering the patient’s physical and psychological ability to tolerate such a device.

## Lengthening

### Humeral lengthening

Humeral lengthening, introduced about 60 years after lower limb lengthening, is a vital procedure in upper extremity reconstructive surgery for multiple hereditary exostosis, achondroplasia, growth arrest, and complications from infections.^5,6^

Despite initial concerns about complications and functional impairment, recent research indicates that increasing humeral length by up to 100% does not significantly raise complication rates, unlike in lower limb lengthening.^2^

**Figure 1.**
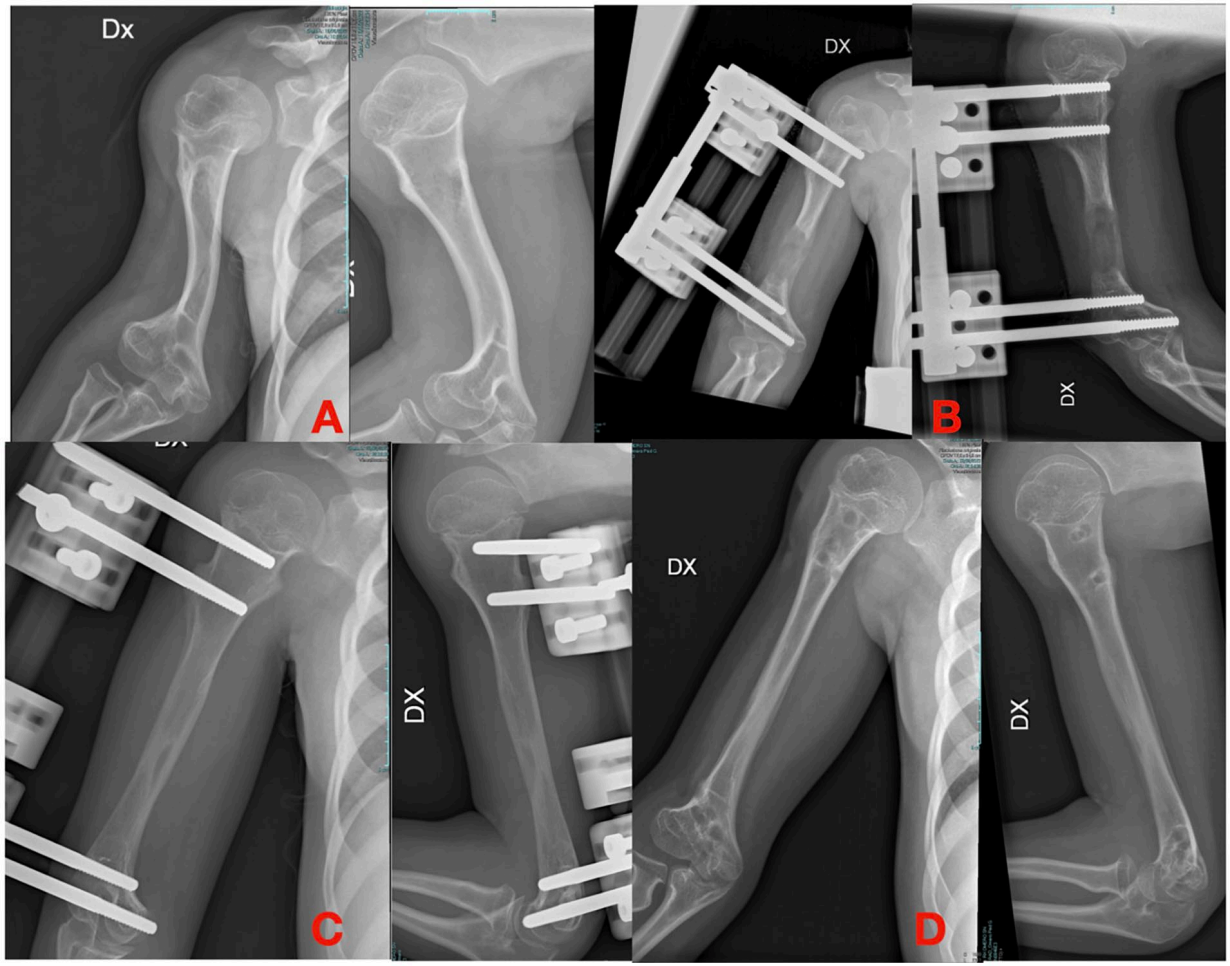
(A) Humeral shortening in achondroplasia. (B) Lengthening with an axial external fixator. (C and D) Final lengthening.

Traditional approaches using external fixators have generally shown positive postoperative outcomes with an acceptable complication rate, as indicated by Yang, Arenas-Miquelez, and Ginebreda (Figure 1).^7–9^

Recent findings suggest comparable effectiveness between circular-framed and monolateral external fixators, with the latter being preferred for their simplicity and improved patient comfort, as observed by Malot and Pawar.^10,11^

However, Laufer et al. observed a notably high incidence of postoperative complications (nearly 50%) particularly in patients with achondroplasia undergoing bilateral humeral lengthening with monolateral external fixators.

Complications included radial nerve palsy, refracture of the regenerated bone, or significant limb length discrepancy, underscoring the importance of careful technique and patient selection in humeral lengthening procedures.^12^

Emerging evidence supports motorized intramedullary nails (MILN) as a safer alternative, reducing complications associated with external fixators such as pin tract infections, and better tolerated by patients, as demonstrated by Morrison et al. in their retrospective analysis of 13 patients.^13^

Galal et al. also advocate for MILN due to its facilitation of earlier rehabilitation and restoration of full shoulder and elbow range of motion, with comparable functional and radiographic outcomes to external fixators in 18 patients.^14^

A systematic review by Lorange et al. emphasizes the efficacy of MILN, particularly using PRECICE and FITBONE systems, in achieving significant humeral length gain with a relatively low complication rate.

Complications mainly related to reduced shoulder range of motion and elbow stiffness, comparable to those with external fixators, albeit slightly higher, with a ratio ranging from 0.5 to 0.8–1. Consolidation time with MILN is similar to external fixators (approximately 27–34 days) but with a notably lower infection rate.^15^

### Forearm lengthening in Multiple Hereditary Exostoses

While isolated osteochondroma seldom causes major symptoms, in multiple hereditary exostoses (MHE) the growth can lead to anatomical deformities of the entire segments: in upper limbs, the forearm is the most affected part according to the classifications of Masada et al.^16^

**Figure 2.**
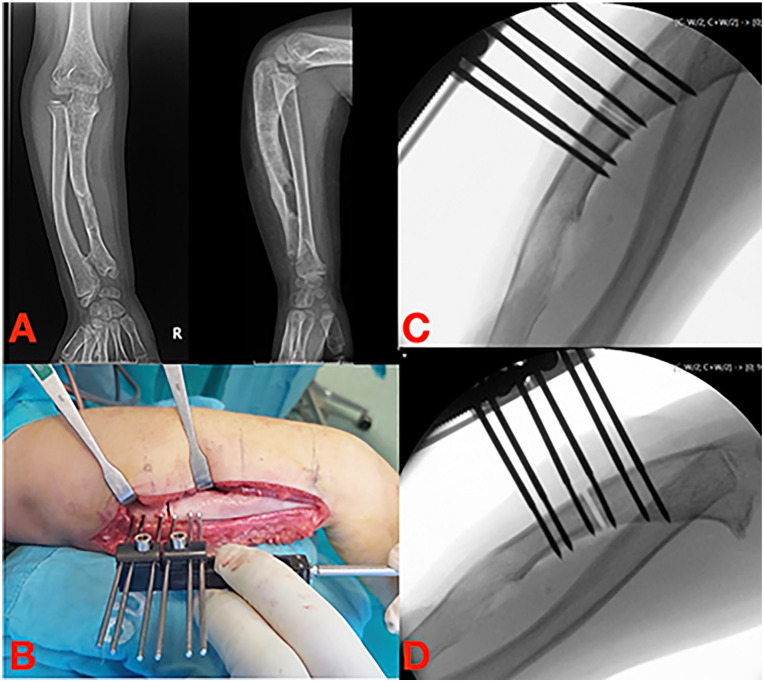
(A) Ulnar shortening in MHE. (B–D) Correction with an axial external fixator and distraction osteogenesis. MHE: multiple hereditary exostoses.

External fixators, especially monoaxial frames, still play an important role in the treatment of forearm deformities in different stages of MHE following Masada classification (Table 1 and Figure 2).^8^

**Table 1.** The three grades of Masada classification in forearm deformities of MHE patients.

MASADA I	MASADA II	MASADA III
Distal ulnar exostoses	Distal ulnar exostoses (type IIb)	Distal radial exostoses
Ulnar shortening	Distal ulnar e proximal radius exostoses(type II a)	Radial shortening
Radial bowing with deviated articular surface	Ulnar shortening	
	Moderate radial bowing	
	Radial head dislocation	

MHE: multiple hereditary exostoses.

Kumara et al., in their series, stated that ulnar lengthening with a mono-planar fixator is the main choice in treating Masada type I and II b; radial shortening osteotomy and ulnar osteotomy may be used, as second-stage procedures, to correct residual deformity.^17^

According to Lu, Masada IIb patients are a progression of type I, and early treatment is recommended; therefore, for achieving radial head reduction, they proposed an osteotomy in the proximal third of the ulna that does not damage the interosseous membrane and proximal oblique cord, with less risk of non-union and delayed union.

Moreover, the authors suggest achieving a 5 mm overlengthening of the ulna to prevent deformity recurrence and ulnocarpal impaction.^18^

Furthermore, many authors highlighted how Masada II is recognized as the most problematic deformity because isolated ulnar lengthening seldom allows the surgeon to obtain radial head reduction.^19–21^

Furthermore, the latter authors have proposed using a ring external fixator which should help in checking that lengthening proceeds at the correct speed and in the right direction.

Cao et al. reported the use of modified ulnar lengthening by Ilizarov external fixators with exostosis excision as a valid method to treat Masada II with radial head dislocation.^22^

Zhang et al. suggested a method of correction for ulnar shortening and radial head dislocation with an Ilizarov frame with a specific hinge positioning method which depends on the locations of the olecranon and the displaced radial head: the declared results seem to be satisficing in such a way the technique can be considered as a valid alternative to other methods.

However, some disadvantages have been reported such as the recurrence of ulnar shortening and bending in younger patients and failure to restore morphological structures of the styloid process.^23^

Hexapod external fixators, the last evolution of Ilizarov’s circular fixator, have been developed to correct, with a single construct and a single operation, multiplanar deformities and achieve length at the same time.

There is a lack of reports in the literature on the use of hexapods in pediatric upper limbs due to the paucity of cases treated with this frame; however, the concept of allowing a simultaneous correction of deviations in three axes with the same device is spreading also for upper limb deformities.

### Radial longitudinal deficiency

Radial longitudinal deficiency is one of the most challenging malformations to deal with: no optimal treatment has been found or shared protocol for different grades in various ages. The real challenge is to treat simultaneously both skeletal malformations (ulnar deviation and radial hypo/aplasia) and soft tissues in the context of a substantial absence of the wrist joint in more severe cases.^24^

Despite the authors being divided on what outcome should be achieved, the ideal treatment should prevent radial deviation recurrence, preserve ulnar growth, and optimize function.

The role of external fixation starts at the beginning of the treatment, allowing the distraction of the soft tissues around the wrist, as a preliminary step to the next surgeries.

Although soft tissue distraction arises from the experience of many surgeons, a recent meta-analysis from Murphy reported low-quality evidence for soft tissue distraction and centralization/radialization as the best option for correction of hand-forearm angle.^25^

**Figure 3.**
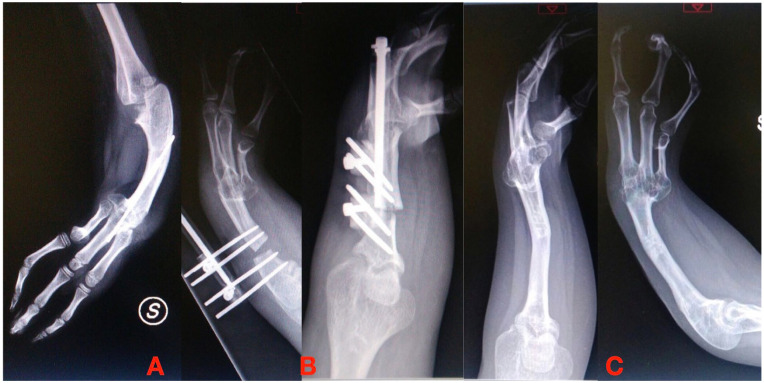
(A) Ulnar shortening in radial longitudinal deficiency. (B) Correction and elongation with the external axial fixator. (C) Final results after 3 cm of lengthening.

External fixators play a major role in adolescents/young adults for forearm global lengthening, especially in bilateral cases in which length is much more important than motion (Figure 3).^24,26^

Both distraction procedures and lengthening can be performed with monoaxial or circular external fixators, but both are not free of complications.

Goreki et al. stated that, with monoaxial construct, it can reach a greater final bone length in patients with single lengthening but stabilization time increases with patients’ age and half of the patients showed complications.^27^

**Figure 4.**
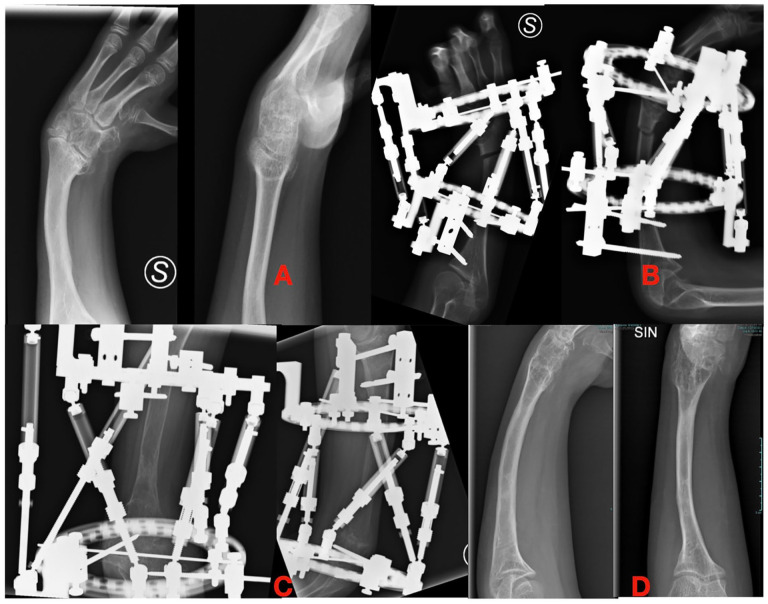
(A) Ulnar shortening and deviation in radial longitudinal deficiency. (B and C) Correction of the wrist axis and elongation with hexapod frame. (D) Final correction.

Hexapod frames are attractive for their property to correct multi-axial deformities as suggested by Launay and Pesenti but literature is still poor nowadays (Figure 4).^28^

### Metacarpal and Phalangeal Lengthening

Brachymetacarpia and brachytelephalangy, as the primary indications for metacarpal and phalangeal lengthening, primarily affect hand aesthetics without significant functional compromise.^29,30^

Reconstructing shortened phalanges and metacarpals remains challenging in hand surgery since it was first described in 1976.^31^

Traditional methods like toe-to-hand transfer are invasive and prone to complications.^32^

Recently, distraction osteogenesis (callotasis) with external fixators (EF) or acute bone lengthening with bone grafts have gained popularity, offering favorable outcomes tailored to patient-specific deformities and expectations.^33,34^

Distraction osteogenesis has shown promising results, improving hand function, metacarpophalangeal joint range of motion (ROM), and cosmetic appearance, with an average metacarpal lengthening of 18.75 mm and consolidation in 38.1 to 49.6 days.^35,36^

However, a 36.6% complication rate exists, including pain, pseudoarthrosis, and joint stiffness.^33^ Recent advancements include the *PAL* (plating after lengthening) technique proposed by Zhang et al., reducing external fixator wear duration, enhancing comfort, and lowering complication rates.^37^ In traumatic digit amputations, innovative techniques such as three-dimensional half-ring frames and Ilizarov mini-fixators offer stable fixation and satisfactory outcomes.^38,39^ Zhu et al. advocate for a mini-Illizarov ring fixator combined with a groin flap for thumb metacarpal lengthening.^40^

A novel approach by Bashour et al. uses a uniplanar internal distractor for metacarpal lengthening, showing success despite a 40% complication rate attributed mainly to device site infections.^41^

## Axis correction

### Cubitus varus deformity

Cubitus varus is the most common late complication of supracondylar humerus fracture in pediatric patients and may also be associated with other distal humerus fracture patterns.^42–44^

Its incidence varies from 9% to 58% and, although it is often described as a purely aesthetic deformity, it can lead to posterolateral rotatory instability of the elbow, changes in elbow range of motion, late ulnar neuropathy, triceps snapping, and progressive varus of the ulna.^44–46^

This condition, resulting from a malunion, consists of a three-dimensional deformity that includes varus in the coronal plane, extension in the sagittal plane, and internal rotation in the axial plane.^47,48^

There are several options, both in terms of osteotomy technique and fixation method, to correct the deformity.^49–54^

External fixation plays its role, with studies demonstrating its advantages and non-inferiority in terms of deformity correction and associated complications compared to other fixation methods such as K-wire or plate and screws fixation.^42,43,55^

In a retrospective study comparing pinning and casting with external fixation after lateral wedge osteotomy for cubitus varus secondary to supracondylar fractures, Shi et al.^55^ even reported that external fixation provided a better functional and aesthetic result with a shorter learning curve.

The different methods of external fixation include the use of monolateral or circular fixators, and methods of acute or gradual correction of the deformity (distraction osteogenesis) with good clinical and radiographic results.^42,43,56–61^

Masquijo, in his retrospective multicentric study of 32 patients, compares lateral closing wedge supracondylar osteotomy fixed with K-wires or with a small monolateral external fixator and states that both techniques are equally effective in correcting the deformity, but stresses that the use of external fixation has the advantage of allowing immediate postoperative mobilization of the elbow and a tendency toward faster recovery of mobility.^42^

The author also emphasizes that with K-wire fixation, loss of correction or under-correction of the deformity is not uncommon and it may be necessary to reposition the K-wires or adjust the osteotomy.

On the other hand, fixation with an external fixator offers greater flexibility in decision-making, allowing intraoperative and immediate postoperative correction/manipulation of the osteotomy and fine-tuning of alignment in different planes.^42,43,55,59,62^

This concept of 3D correction is shared by other authors and is described as being able to improve muscle activity and joint motion of the affected elbow, as well as providing a better cosmetic result.^43,63,64^

In a study carried out by Tang et al.^43^ with 35 patients comparing the clinical and radiological results of lateral closing-wedge osteotomy with either internal fixation (4-hole 3.5 mm locking plate) or monolateral external fixation (AO small fixator), he concludes that both techniques provide satisfactory functional and aesthetic results.

The author emphasized that the external fixation technique was associated with shorter operating times, a smaller scar, and lower costs.

Moreover, he highlighted that, unlike the plate fixation technique, in his study there were no cases of loss of correction or elbow mobility with the external fixation technique.^43^

The use of external fixation also allows progressive three-dimensional correction of the deformity using the compression-distraction osteogenesis method with circular fixators such as the Ilizarov or Taylor Spatial Frame.^59,65,66^ In his retrospective study of 32 patients, Agrawal highlighted the advantages of this gradual correction method but noted that it is not without complications such as overcorrection or pin-site infection.^59^

Agrawal also pointed out that although this technique allows for postoperative correction of the deformity and active mobilization of the elbow, it has a long learning curve and therefore requires meticulous planning and respect for Ilizarov’s basic principles.^58^

Belthur added that gradual correction of the deformity with an external fixator has the potential benefit of reducing the risk of ulnar nerve damage, but that it is important to explain to parents that the procedure requires a longer treatment time and to discuss patient and family compliance with this method.^63^

Other described complications associated with external fixation are under-correction, refracture, joint stiffness, infection, unsightly scars, or iatrogenic, late ulnar, or radial nerve palsies.^42,43,55–60,66^ The use of external fixation with K-wires and methyl methacrylate is also an option described in the literature.^60^

Acar’s retrospective study of 14 patients who underwent lateral closing wedge osteotomy stabilized with multiplane K-wires and methylmethacrylate found that lateral bone-cemented external fixation was a practical, effective, reliable option with good patient tolerability and lower cost than other external fixation systems.^60^

### Cubitus valgus deformity

Cubitus valgus deformity frequently arises due to non-union or lateral physeal arrest of the humerus, commonly associated with lateral humeral condyle fractures.

These fractures represent the second most common type of elbow fracture in children and adolescents, surpassed only by supracondylar fractures.^67-69^

Cubitus valgus deformity can lead to lateral elbow instability and stiffness, as well as tardy ulnar nerve palsy.^70^

The correction of cubitus valgus through different corrective osteotomies combined with anterior transposition of the ulnar nerve is a widely practiced surgical approach.

However, there is limited literature on the use of external fixation techniques for this deformity, with most studies focusing on traditional osteotomy methods.^71–73^

The first recorded use of an external fixator for the correction of residual elbow deformities was described in a 2005 in vitro study, which reported favorable results when tested on cadaveric models.

This early research demonstrated the potential efficacy of external fixation for managing such deformities, laying the groundwork for further exploration in clinical settings.^74^

Subsequent research has continued to investigate the use of external fixation techniques, including a notable 2007 study led by Kamineni and colleagues.

Their findings indicated that a lateral dynamic elbow external fixator was effective in maintaining varus displacement; however, they observed that valgus displacement was more vulnerable to additional loading, particularly when associated with damage to the medial soft tissues.

This highlights the fixture’s limitations in managing valgus deformities under specific biomechanical conditions.^75^

However, a 2012 study by Al-Sayyad reported favorable outcomes in the management of elbow deformities using a modern multiplanar external fixator, the Taylor Spatial Frame (TSF).

This device proved effective in achieving precise corrections, particularly in complex cases, demonstrating its utility as a reliable option for treating upper extremity deformities.^76^

A recent retrospective review by Rao and Cohen, conducted in 2021, assessed the outcomes of treating chronic elbow instability using static external fixators in 27 patients.

Their findings indicated a favorable result in 95% of cases, with follow-up data extending to nearly 6 years, demonstrating the long-term efficacy of this treatment approach for managing persistent elbow instability.^77^

Elbow instability, a common complication following dislocations and fractures, continues to present a significant challenge in treatment; in recent studies, a frequently employed surgical intervention involves the use of a hinged external fixator.

This technique has been supported by multiple studies, demonstrating it as a reliable treatment method with consistently favorable outcomes and an acceptable rate of complications.

Various research groups have validated its efficacy, particularly in cases where conventional methods prove insufficient for maintaining elbow stability post-injury.^78,79^

On the other hand, known complications of external fixators, such as the prolonged duration of the in situ treatment, coupled with the associated discomfort, make it an unfavorable option for some patients.^80^

Complex supracondylar fractures can also lead to the development of cubitus valgus.

These fractures are generally treated with closed reduction and percutaneous fixation.

A biomechanical study by Li et al. evaluated various treatment approaches for pediatric supracondylar fractures.

The study found that external fixators, particularly the Orthofix model, provided more stable osteosynthesis and resulted in fewer soft tissue complications when compared to K-wire configurations, making it a preferable option for maintaining fracture alignment and reducing postoperative issues.^81^

## Surgery related to complications

### Missed Monteggia

Monteggia fracture is a traumatic condition that affects the forearm characterized by the association of an ulna fracture with a dislocation of the radial head.^82^

The first description was attributed to Dr. Giovanni Battista Monteggia,^83^ in 1814, but only many years later, in 1967, Dr. Jose Luis Bado published a monograph on Monteggia’s injury recognizing four different types according to the site of ulnar fracture and the direction of radial head dislocation.^84^

Despite the efforts of many authors who contributed, over the years, to increase the comprehension of the different aspects characterizing Monteggia’s injury, it remains a challenge for orthopedists, especially due to the high rate of missed cases which can lead to serious consequences due to the difficulty in managing the chronic condition.

The treatment goals, universally recognized in acute cases, are to obtain an anatomic reduction of the ulnar fracture, a congruent radio-capitellar joint, and to maintain ulnar length and stability.

The last point can be achieved with different devices; usually, in the pediatric population, endomedullary elastic fixation or Kirschner wires are the first choice besides plates and screws are the best option for adults.

As regards missed or chronic Monteggia, there is no consensus about the optimal treatment.^85^ Even if there are no clear criteria to define this condition because a strict temporal criterion is not useful and could be advisable to consider ulnar healing as a parameter for the choice of treatment (closed reduction attempts versus ulnar lengthening needing to reduce radial head).

According to the authors,^85^ indeed, the debated points scheduling treatment are the need to:

– lengthening of the ulna, gradually or immediately, with a flexion-angulation osteotomy
– lead the radial head to be relocated, gradually or immediately
– associate, eventually, an open reduction to clean the radio humeral space
– repair/reconstruct the annular ligament
– choice of some devices to maintain the stability during healing process.

From a recent literature review,^85^ external fixators have been used, especially to fix the ulnar osteotomy although synthesis with plates and screws is much more widespread; in the analysis of 395 ulnar osteotomies, only 14% were fixed with an external device (mono-axial or circular external fixator).

**Figure 5.**
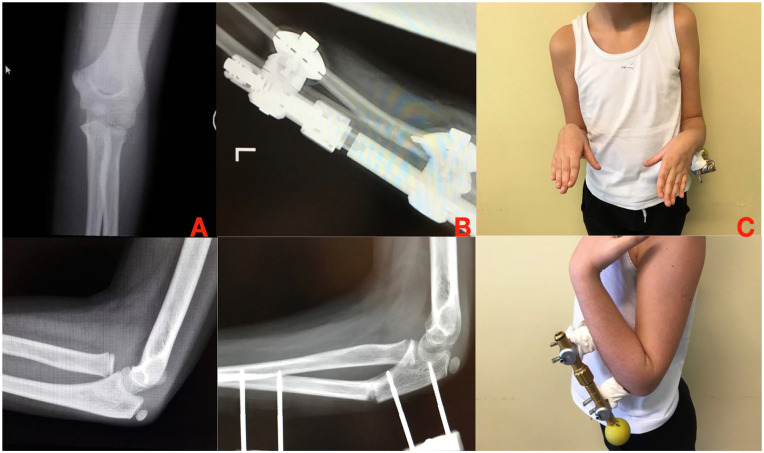
(A) Missed Monteggia 2 months after trauma. (B and C) Open radial head reduction and ulnar flexion osteotomy with an axial external fixator.

In the majority of the cases treated with mono-axial fixators, they were applied as a fixation device after an angulation/flexion osteotomy of the ulna, associated with an immediate reduction of the radial head (Figure 5).

The osteotomy was located, in 33 cases,^86^ at the diaphyseal-metaphyseal junction of the proximal segment, in 3 cases,^87^ at the proximal diaphyseal level, and 5 cases^88^ at the proximal third of the ulna or the fracture site.

The fixator, dual socket^86^, the standard model with a three-dimensional joint connected^87^ or Ilizarov mini-fixator,^88^ was applied to the ulna with two proximal and two distal screws or wires, in the dual socket with the most proximal screw as close as possible to the coronoid process to give a more stable construct.^86^

Only in 1 case, a closed reduction of the radial head was achieved^87^; in other cases, an open reduction was performed to clean the joint space.

Fewer cases used the mono-axial fixator with the aim of ulna gradual lengthening for progressive radial head reduction; in 1 case, the reduction was achieved with a flexion osteotomy of 20° associated with the removal of scar tissue surrounding the radial head, the fixator was used to lengthen the ulna during callus formation for a total of 7 mm^89^; in 10 cases, after the osteotomy, a gradual lengthening and progressive angulation were obtained with the device but only in 1 case a closed reduction of the radial head was achieved.^90^

Mono-axial fixator was used, also, as a distractor.

In 13 cases described by Wang, the surgical strategy consisted of temporary external fixator-assisted ulnar osteotomy with distraction, open reduction of the radial head, and ulnar osteosynthesis with plate and screws after achieving a stable reduction in pronation with the removal of the external device.^91^

In the 4 cases treated with the circular fixator, the goal was to achieve the reduction with a gradual length of the ulna.

A single half ring with 4 threaded pins was applied in the proximal ulna and a distal circular ring was placed in the diaphysis with the osteotomy performed between them, at the diaphyseal-metaphyseal junction of the proximal ulna.^92^

After 7 days of latency was started the correction, according to the program schedule, until the radial head reached the correct position; the frames were removed after a mean of 3.5 months, with a gain of 2 cm of length.^92^

The advantage of using an external device, with respect to an internal synthesis, is that it is a more versatile instrument that can be used for the synthesis only, in case of performing an immediate reduction (closed or open) of the radial head with a flexion angulation osteotomy of the ulna, or for a gradual correction that, with a progressive flexion and lengthening of the ulna, led the radial head to relocate spontaneously.

An immediate correction could be debating the choice between plates and screws or mono-axial external fixators for the synthesis, while the progressive correction with mono-axial or circular fixators is theoretically less invasive, avoid an extensive approach with wide exposure of the ulna, enable a controlled reduction, and overcome tissues contractures. In the literature review, however, a closed reduction of the radial head was achieved only in 2 cases treated with mono-axial fixators but in all cases treated with circular external fixators according to Ilizarov principles.

The main disadvantage of external fixation, especially for circular constructs, is the need for a more trained surgeon, the clutter of the device for many months^91^ that can be noisy for patients and parents, and the need to understand previously if an anatomic reduction of the radial head can be performed without problems.

## Conclusions

The indications for the use of external fixators to correct deformities of the upper limb remain much more limited than those of the lower limb.

Monoaxial fixators retain a prominent role for skeletal lengthening in all the segments examined, although their role could be outclassed by the motorized intramedullary nails, especially for humeral lengthening.

Hexapod systems are like to represent the future for correction of multiplanar forearm deformities; however, multicenter studies on larger series will be necessary to better validate their applications and advantages.

## Supplemental Material

sj-pdf-1-cho-10.1177_18632521251327127 – Supplemental material for External fixators and lengthening systems in pediatric upper limb Supplemental material, sj-pdf-1-cho-10.1177_18632521251327127 for External fixators and lengthening systems in pediatric upper limb by Nunzio Catena, Chiara Arrigoni, Marcos Carvalho, Ida Matic and Sebastian Farr in Journal of Children’s Orthopaedics
